# A Safety-II Perspective on Organisational Learning in Healthcare Organisations: Comment on "False Dawns and New Horizons in Patient Safety Research and Practice"

**DOI:** 10.15171/ijhpm.2018.16

**Published:** 2018-02-18

**Authors:** Mark Sujan

**Affiliations:** Warwick Medical School, University of Warwick, Coventry, UK.

**Keywords:** Safety-II, Resilience Engineering, Patient Safety, Work-As-Done, Organisational Learning

## Abstract

In their recent editorial Mannion and Braithwaite provide an insightful critique of traditional patient safety improvement efforts, and offer a powerful alternative vision based on Safety-II thinking that has the potential to radically transform the way we approach patient safety. In this commentary, I explore how the Safety-II perspective points to new directions for organisational learning in healthcare organisations. Current approaches to organisational learning adopted by healthcare organisations have had limited success in improving patient safety. I argue that these approaches learn about the wrong things, and in the wrong way. I conclude that organisational learning in healthcare organisations should provide deeper understanding of the adaptations healthcare workers make in their everyday clinical work, and that learning and improvement approaches should be more democratic by promoting participation and ownership among a broader range of stakeholders as well as patients.

## Introduction

In their insightful editorial Mannion and Braithwaite^1^ consider “false dawns and new horizons” for the still relatively young patient safety discipline. Their contribution is a critical analysis of the early and current patient safety research efforts, which have been frequently overly optimistic, and which have been, at times, simply too naïve. The outlook they provide has the potential to radically change the way we look at patient safety. Rather than framing safety as the absence of harm (referred to as Safety-I), which comes with negative connotations such as human error, they offer a vision wherein safety (Safety-II) is described in the affirmative – as the ability to navigate successfully the stresses and tensions present in any modern day complex system.^2^ This is a fundamental shift in perspective, which has gained increasing popularity in the traditional safety-critical industries,^3,4^ and which we are now starting to embrace in healthcare.^5-9^

In this commentary, I explore how the Safety-II perspective points to new directions for organisational learning in healthcare organisations. Right from the start of the mainstream patient safety movement, policy-makers identified organisational learning as a key strategy for improving the safety of care.^10^ This was emphatically reiterated in the Berwick report (suitably entitled “A promise to learn – a commitment to act”) following the investigation into the failings at Mid Staffordshire NHS Foundation Trust. The report emphasised that the National Health Service (NHS) needed to become a system that was devoted to continuous learning and improvement.^11^ Even though there has been sustained focus on improving patient safety through organisational learning, healthcare organisations are struggling to generate useful learning and to translate learning into meaningful and sustainable improvements in practice.^12-14^ I argue that this is at least in part due to the focus of organisational learning as practised in healthcare organisations on adverse events and incidents, ie, on things that have gone wrong. This approach to learning only considers the few extraordinary situations, where a system has broken down, ie, we are only seeing half the story, at best.^15^ A Safety-II approach to organisational learning enables us to learn about why, most of the time, things go right, ie, the manifold adaptations in the system that prevent everyday disturbances and disruptions from becoming everyday catastrophes.^16^ I conclude that healthcare organisations could use the Safety-II perspective to create a more positive, inclusive and ultimately more effective learning environment for improving patient safety.

## Healthcare Organisations Fail to Learn

The literature on organisational learning is heterogeneous and broad, with no universally agreed definition.^17^ Organisational learning has been characterised as a continuous cycle of action and reflection, which can take place at different levels (individual, group, organisation or even business sector).^18^ Organisations have a range of learning processes at their disposal, both internal and external.^19^ However, in healthcare organisational learning has been implemented relatively narrowly. Healthcare organisations attempt to learn mostly from retrospective analysis of failures and incidents in order to prevent such events from happening again. In the NHS the National Reporting and Learning System (NRLS) was set up to collect and to aggregate data about incidents at a national level. NRLS was established in 2003, and to date over 4 million incident reports have been submitted to the system. This number already far surpasses the roughly 1.25 million incident reports that are contained within the US Aviation Safety Reporting System (ASRS) – which was established in 1976. As far as the reporting of incidents is concerned, it could be argued that NRLS has been highly successful. But what about the learning from these vast numbers of incident reports?

Longitudinal studies suggest that there is a lack of evidence that care has become any safer over the years.^20-22^ In addition, many commentators have provided critical analyses of incident reporting systems and other retrospective analysis techniques, such as root cause analysis.^23-27^ Criticisms that have been raised include inadequate feedback to people contributing incident reports, the lack of visible improvements to clinical practice, the development of weak improvement interventions, and the use of organisational learning approaches as tools for management rather than for improvement. In addition, incident reporting and root cause analysis can be perceived as contributing to the existing blame culture^10^ because there is a temptation to focus on what individuals did wrong.^13^ The breadth of these criticisms has prompted some to argue that these tools are part of the problem of the lack of progress on patient safety, rather than part of the solution.^25,28^

Another important approach to encourage learning from experience in healthcare organisations is the use of indicators and trend data.^29^ Frequently, indicators tend to be lagging indicators, ie, outcome measures, such as the number of pressure ulcers or patient falls. Other indicators can be regarded as leading indicators and reflect aspects of healthcare processes. Examples include the percentage of patients for whom a venous thromboembolism (VTE) assessment has been undertaken, and the percentage of patients identified as at risk who have received VTE prophylaxis. I do not discuss further the use of indictors in the critique below, but mostly indicators are measures derived from the Safety-I paradigm. Hence, they provide information about adverse outcomes and sources of failure and unreliability, rather than about the extent to which a system is able to anticipate and to adapt to changes and disturbances.^30^

## Organisational Learning in Healthcare From a Safety-II Perspective

Looking at organisational learning for improving patient safety from a Safety-II perspective, I want to focus specifically on two aspects of current approaches that are fundamentally flawed: we are learning about the wrong things, and we are learning and improving in the wrong way.

Mannion and Braithwaite suggest that patient safety research and practice should be concerned with what goes right rather than what goes wrong, ie, the focus should be on work-as-done on an everyday basis. The focus of many current organisational learning approaches in healthcare, however, is specifically on adverse events and incidents. In this way, the learning is about what went wrong, what kinds of mistakes were made, and which barriers and safeguards have failed. While such insights can be useful, they are hardly adequate by themselves to provide a good understanding of safety in complex adaptive systems. Studies undertaken under the umbrella of resilient healthcare demonstrate that it is the ability of healthcare workers and clinical systems to adapt and to make dynamic trade-offs, which enables them to provide safe care in the face of disturbances, ambiguities, tensions and contradictions, and competing organisational priorities.^8,31-36^ Often, such studies provide rich ethnographic accounts of the adaptive strategies that healthcare workers adopt, such as sacrificing and reformulating goals, offloading of demands, pulling in extra resources, and trading risk based on subjective assessments of the needs of a clinical situation. The aim of learning and improvement should be to appreciate the positive contribution such adaptations make, and to identify ways in which this adaptive capacity can be strengthened.^37^ Unfortunately, studies of the practice of root cause analysis in healthcare suggest that the opposite is currently happening – solutions that are based on “learning” from these analyses tend to focus on training, the development of protocols, and reminders to staff to follow the rules.^25^ Such solutions are reinforcing work-as-imagined rather than fundamentally challenging assumptions about what keeps patients safe.^38^

A corollary to the focus on work-as-done is the need to make organisational learning in healthcare more democratic. When interviewing healthcare workers about reporting, learning and improving patient safety I was struck by how little staff who contribute to incident reporting were told about how the process works.^39^ A common expression used was that incident reports “disappeared into a black hole.” Staff were largely unaware of who would be looking at the reports, and whether or how learning would be generated. Incident reporting systems and root cause analyses are usually owned and overseen by risk management departments or patient safety officers, with little ownership by frontline healthcare workers. On the other hand, many of the actual improvement efforts appear to take place in less formal settings, such as lunchtime working groups or interdepartmental teams that have formed around a common improvement objective. In the literature the importance of these communities of practice has been recognised.^40^ It has also been suggested that the practice of organisational learning might be enhanced through an open political system, ie, a democratic approach where people have the psychological safety to speak up and create learning in dialogue and through constructive criticism of ideas and views.^41,42^ However, healthcare organisations have failed to embrace such efforts as part of their formal strategies for harnessing learning and improving patient safety. Organisational learning in healthcare is limited by this dichotomy between formal risk management efforts aimed at bringing work-as-done in line with work-as-imagined, and frontline efforts directed at improving everyday clinical work. There is an urgent need to appreciate these latter efforts, and to embed them within the formal organisational learning strategy.

## New Directions

Examples of promising innovations in organisational learning can be found both within the increasing body of literature on resilient healthcare as well as beyond. A number of recent approaches focus on purposefully monitoring the adaptations (work-as-done) healthcare workers make to procedures and protocols (work-as-imagined).^43,44^ The aim of these approaches is not to identify violations and to constrain behaviour, but rather to understand the organisational context and the tensions within clinical systems. Healthcare workers are encouraged to provide feedback about procedures and protocols, and to document the adaptations they need to make. This can feed into a process of reflection about work-as-imagined and sharing of best practice. While this process is arguably still limited in terms of the solutions it generates (protocols and procedures), it supports adaptations through resilient procedures, ie, through providing different options and decision criteria to healthcare workers instead of prescribing a single, assumed best practice.^45,46^

In my own work, I have used staff narratives about their daily “hassles” to document not only the manifold problems healthcare workers encounter every day, but also how they adapt to these problems and how they continue to deliver safe care.^47^ This process of “hassle reporting” is very simple, and can be empowering when healthcare workers are encouraged and supported by senior management to develop solutions within their own work environment. The focus on hassle and on how people deal with it avoids potentially threatening notions of human error and patient harm, and should thus reduce fear of repercussions. In this way, the process of learning can be geared much more easily towards improvement and less towards assignment of culpability.

Similarly, an approach to learning from experience based on “excellence reporting” has been developed at Birmingham Children’s hospital, and is gaining popularity in the NHS.^9^ This Learning from Excellence (LfE) approach encourages members of staff to report episodes of good practice that they have observed. This approach can provide learning about useful adaptations, and it can contribute to enhancing staff morale and workplace culture.^9^

Interesting examples of how to make learning more democratic (ie, involve and give a voice to a broader range of stakeholders) can often be found in customer focused domains, such as retail and tourism, where there are many examples of popular rating and feedback sites that give a voice to customers. As healthcare is aiming to become more patient-centred, we are now seeing similar initiatives changing the way organisations can and should learn about the quality of care they provide. One such example is Care Opinion (https://www.careopinion.org.uk/), which is a not-for-profit organisation running a web site and database, where patients can describe experiences with the care they have received. Healthcare organisations can (for a fee) access this feedback and use it as an input to their improvement efforts.

In her role as citizen-patient, Carolyn Canfield reflected on the democratisation of healthcare, and the contribution of patients to resilient healthcare, and concluded insightfully: “Inviting past, present and future patients and their carers to grow from disempowered outsiders to become active participants will enhance health care’s need to comprehend quickly, flexibly and sensitively those components of care that only care recipients can perceive.”^48^

Arguably, there is at present still a lack of studies that provide rigorous evidence about the benefits of adopting Safety-II approaches to support organisational learning in healthcare, or more generally of the contribution of Safety-II to enhancing patient safety. The research focus has largely been on describing how individuals, teams and organisations anticipate and adapt successfully to changes and disturbances. While there exist experience reports describing practical implementation issues of Safety-II programmes,^9,14,47,49,50^ further work is required to understand and to assess how Safety-II programmes can be implemented and improve patient safety.

## Conclusion

The importance of effective organisational learning to improve patient safety can hardly be overstated. The main approaches to organisational learning in healthcare based on incident reporting and root cause analysis have had limited success, and have attracted frequent criticism. While such approaches that attempt to learn from adverse events and incidents can be useful, complementary approaches are urgently needed. Building on the Safety-II perspective it might be possible to devise novel approaches to organisational learning in healthcare organisations that can provide richer insights into how complex adaptive systems deliver safe care; and that increase participation in and ownership of organisational learning among healthcare workers and patients.

## Ethical issues

Not applicable.

## Competing interests

Author declares that he has no competing interests.

## Author’s contribution

MS is the single author of the paper.
